# Stromal Genes Add Prognostic Information to Proliferation and Histoclinical Markers: A Basis for the Next Generation of Breast Cancer Gene Signatures

**DOI:** 10.1371/journal.pone.0037646

**Published:** 2012-06-18

**Authors:** Dwain Mefford, Joel Mefford

**Affiliations:** 1 Trinidad, California, United States of America; 2 Department of Epidemiology and Biostatistics, University of California San Francisco, San Francisco, California, United States of America; Health Canada, Canada

## Abstract

**Background:**

First-generation gene signatures that identify breast cancer patients at risk of recurrence are confined to estrogen-positive cases and are driven by genes involved in the cell cycle and proliferation. Previously we induced sets of stromal genes that are prognostic for both estrogen-positive and estrogen-negative samples. Creating risk-management tools that incorporate these stromal signatures, along with existing proliferation-based signatures and established clinicopathological measures such as lymph node status and tumor size, should better identify women at greatest risk for metastasis and death.

**Methodology/Principal Findings:**

To investigate the strength and independence of the stromal and proliferation factors in estrogen-positive and estrogen-negative patients we constructed multivariate Cox proportional hazards models along with tree-based partitions of cancer cases for four breast cancer cohorts. Two sets of stromal genes, one consisting of *DCN* and *FBLN1*, and the other containing *LAMA2*, add substantial prognostic value to the proliferation signal and to clinical measures. For estrogen receptor-positive patients, the stromal-decorin set adds prognostic value independent of proliferation for three of the four datasets. For estrogen receptor-negative patients, the stromal-laminin set significantly adds prognostic value in two datasets, and marginally in a third. The stromal sets are most prognostic for the unselected population studies and may depend on the age distribution of the cohorts.

**Conclusion:**

The addition of stromal genes would measurably improve the performance of proliferation-based first-generation gene signatures, especially for older women. Incorporating indicators of the state of stromal cell types would mark a conceptual shift from epithelial-centric risk assessment to assessment based on the multiple cell types in the cancer-altered tissue.

## Introduction

After a decade of development and early success, the enterprise of building gene-based classifiers for breast cancer risk assessment is entering a period of review and integration/consolidation [1], [2], [3], [4], [5], [6], [7]. Gene-based studies have delivered insights into the complexity of breast cancer, especially the recognition that breast cancer consists of multiple distinct diseases at the molecular level [8], but to date the process of translating these insights into clinical practice has been halting and incomplete [9], though gene-based diagnostics have demonstrated sufficient value as aids to the prognostication of early breast cancer for estrogen-positive patients to be cleared for clinical use by the FDA [10], [11] and, in the case of the OnctotypeDX 21-gene Recurrence Score, to be approved by ASCO and NCCN [12]. Compared to available indices and scores such as Adjuvant! Online and the St. Gallen guidelines, gene signatures have demonstrated greater accuracy in discriminating “good” from “poor” prognoses, at least for estrogen-positive patients within a near-term (five year) time frame, though the improvement over optimized clinicopathological measures or over indices such as the Nottingham Prognostic Index may be modest [13], [14], [15].

First-generation gene signatures, most prominently the Amsterdam70-gene, Rotterdam 76-gene, and Genome Grade Index (GGI) [16], [17], [18] are driven principally by proliferation and cell-cycle genes [19], [20], [21]. In fact, several of the gene signatures become more sensitive and specific when all but the proliferation genes are removed [22]. Moreover, in a proof of concept Haibe-Kains and colleagues have shown that the signal in a single proliferation gene, *AURKA*, performs nearly as well as several of the best-studied gene signatures despite the fact that they are comprised of dozens or hundreds of genes [23]. Since proliferation genes are mostly up-regulated for estrogen-negative tumors, they may not exhibit the variance needed to discriminate low risk from high risk patients in this subpopulation [22], [24], [25].Gene signatures that appear to apply to both estrogen-positive and estrogen-negative subsets, for example the 3D model-based signature developed by Martin et al. [26], [27], rely on separate subsets of genes, one prognostic for estrogen-positive samples, the other for those that are estrogen-negative.

In addition to prognostic and predictive gene signatures, the principal contribution of microarray-based studies has been the delineation of breast cancer subtypes. A consensus has emerged that at least three subtypes exist, one that expresses markers for estrogen receptor α, but not for HER2, one that expresses HER2, and a residual subtype that expresses neither [28]. General agreement as to how these subtypes might be further elaborated has yet to emerge, though a strong case has been made that the estrogen receptor-positive or luminal subtype can be further divided by the expression of proliferation genes or biomarker for proliferation such as Ki67 [18], [29].

Against this backdrop, among the efforts to refine and extend gene-based classifiers, two lines of research stand out. One involves efforts to define subsets of estrogen-negative or HER2-positive on the basis of sets of immune-related genes [30], [31], [32], [33]. The second involves stromal signatures [34], [35], [36], [37], some of which appear to be driven, again, largely by the proliferation signal. Important exceptions to this include West et al.’s Desmoids-Type Fibromatosis (DTF) signature [38], [39], [40] and a stromal metagene derived by Bianchini et al. [41]. Prominent on both of these gene lists are collagen-related genes including *SPARC, CSPG2, FBLN2, FBN1*, and type-I, type-III, and type-VI collagens. The DTF signature was devised as a proof of concept for a larger, on-going program that exploits the mono-cellular property of soft tissue tumors to inductively define subtypes (or states) of fibroblastic stroma cells [38], [39], [42]. It is significantly prognostic of increased survival for breast cancer [40].

From an earlier investigation of the stromal sets as prognosticators of survival there is evidence that the stromal genes add information to estrogen and proliferation expression and to such clinical measures as lymph node involvement [43]. Specifically

- The stromal set consisting of *DCN* and *FBLN1* adds prognostic value for near-term events (<2.5 years) for both lymph node-positive and lymph node–negative patients.
- This stromal-decorin set adds substantial prognostic value for near-term events for samples both up-regulated and down-regulated on proliferation genes.
- A second stromal set, in which *LAMA2* figures prominently, may add prognostic value to both estrogen-positive and estrogen-negative cohorts, though for this dataset the number of estrogen-negative samples and events is very small [44].

These initial findings suggest, on the one hand, that the stromal sets might possibly improve the performance of first-generation, proliferation-based gene signatures. On the other hand, these genes might be incorporated into the definitions of sub-subtypes in a future breast cancer typology. To establish whether the stromal gene sets add significant independent information to the proliferation signal and to clinical descriptors, we built Cox proportional hazards models to investigate disease free survival as functions of combinations of these factors. We fit these models to the data as a whole and, separately, to estrogen-positive and estrogen-negative patients for each of the four breast cancer datasets listed in Table 1. In addition to the Cox regressions, we searched for prognostic gene sets using a visualization device that in effect builds decision trees from partitioned heatmaps of expression values. The predictors in the Cox models, and the partitioned gene sets in the figures, include three sets of genes (proliferation, stromal-decorin, and stromal-laminin), along with clinical measures, principally lymph node status and tumor size.

**Table 1:** Datasets.

Dataset	Samples^a^	ER+/ER−b	Age^c^	LNN/LN+^d^	Size^e^	Grade^f^	Endpoint^g^	Data source	rf
Uppsala	251/236	202/34	64+/−14^h^	158/84/9	22+/−13^h^	69/126/54/2	BCSS	SE3494	[44]
Mainz	200/200	156/44^i^	60+/−12^i^	200/0	21+/−10^h^	29/136/35/0	DMFS	GSE11121	[32]
San Francisco	118/117	74/43	51+/−15^h^	71/66	27+/−14^h^	14/46/65	DMFS	E-TABM-158	[50]
Stockholm	159/159	130/29	56+/−14^h^	94/60	22+/12^h^	28/58/61/12	BCSS	GSE1456	[49]

a Number of samples/Number of samples with survival data.

b Estrogen receptor status.

c Median age in years; mean age for Mainz.

d Lymph Node status: LNN/LN+/unknown.

e Average tumor size in cm.

f Histological Grade: 1/2/3/unknown.

g Endpoint: Breast Cancer Specific Survival, Distant Metastasis Free Survival.

h source = [57].

i As reported in [32], but not available in GSE11121.

### Estrogen Gene Set

In the effort to determine whether the stromal signal was substantial and independent of both estrogen and proliferation when adjusting for clinicopathological measures, we applied univariate and multivariate Cox regression using stepwise backward elimination with a inclusion cutoff of *p* = 0.10. In two of the four datasets estrogen receptor status is available for each patient. For the MAINZ and STOCKHOLM cohorts only the total number of estrogen-positive and estrogen-negative samples is reported. In those cases, as a surrogate for estrogen-status we used a dichotomous variable based on expression levels for the genes in the estrogen gene set discovered by the partitioning algorithm. That estrogen gene set, which includes *ESR1*, *GATA3, CA12, JMJD2B, FOXA1, TBC1D9, SLC7A8* closely matches a number of lists reported in the literature, including, for example, the Sensitivity to Endocrine Therapy (SET) index, a list of genes whose expression correlates with *ESR1* [45].

### Proliferation Gene Set

To render proliferation as a continuous variable in the Cox proportional hazards models we used a set of proliferation genes we found previously [43]. The genes in this set coincide closely to lists of proliferation and cell cycle related genes that have been reported in several microarray-based studies of breast cancer [18], [22], [25], [46], [47]. Genes prominent in this set include: UBE2C, TPX2, FOXM1, BIRC5, TOP2A, and AURKA aka STK6. Of the forty-one distinct genes in the proliferation set, all but four belong to the list of 97 genes that define the GGI index (while one of the remaining four is found in the less stringent version of the GGI list) [18]. The genes in our proliferation set also constitute a proper subset of a second proliferation list developed by Wirapati et al. [22] using a supervised method.

### Selecting Two of Four Stromal Sets as Representative

As previously reported, there are at least four stromal gene sets [43]. Although formal tests for separation indicate that these sets induce distinct patterns of partitions of breast cancer samples into subgroups, a case can be made that the four can be reduced to two pairs of sets, and, consequently, can be represented by just two of the original sets. Specifically, the ordering induced on the breast cancer samples by expression levels for the genes in the large collagen set, which closely resembles West et al.’s DTF signature, is quite similar to the ordering on the samples induced by the small set consisting of only decorin and fibulin-1. Similarly, the two remaining stromal sets induce similar orderings on breast cancer samples when expression levels across the genes are ranked. These two gene sets characterized, respectively, by *LAMA2* and *CAV-1*, might be jointly represented by the laminin set, which is comprised of: *LAMA2, IGF1, C10orf56, MFAP4, COL14A1, ZNF423,* and *ABCA8*. (It should be noted that unlike gene lists assembled on the basis of a correlation, the ordering of the genes is not informative, reflecting only the ordering of the probe sets on the Affymetrix HG-U133A platform. Also, since each gene in a particular set induces essentially the same ordering and partitioning on the breast cancer samples, proper subsets of a gene set may be functionally substituted for the set as a whole.).

Beyond the convenience of small size, a justification for choosing the stromal-decorin and stromal-laminin sets is that they are, at least in univariate models, among the most prognostic of increased survival [43]. A second justification concerns possible biological relevance due to biological function and cell type. That is, it may be the case that these two sets, though related in the pattern of partitions expressed, may reflect changes in the state of aberration of two different cell types. The loss of decorin and laminin expression may be part and parcel of the de-differentiation of myoepithelial and myofibroblast cells in the case of decorin, and of myoepithelial cells, in the case of laminin. The loss of expression of these decorin and laminin gene sets could signal the extinction of the myoepithelial cell layer and the de-differentiation of stromal fibroblasts (reactive stroma). These sets may, therefore, potentially function as markers for the state of cell types in the stromal compartment. They might serve as indicators of the extent or degree of tissue change reflecting progression of the disease. If this is the case, then the choice of the stromal-decorin and stromal-laminin sets could be justified by their potential roles as indicators of the aberrant state of at least two cell types within the cancer-affected tissue.

## Results

### Uppsala

The UPPSALA dataset as reported in [44] is a population-based cohort of 251 consecutively presented breast cancer patients obtained from Uppsala county, Sweden, in the years 1987 to 1989. The Affymetrix HG-U133A expression data is available at Gene Expression Omnibus (GEO) [48] as series GSE3494, along with clinical descriptions, including Breast Cancer Specific Survival events and times for 236 of the samples.

Using Kaplan-Meier product limit estimations we previously reported that the proliferation and four stromal sets are significant univariate predictors of disease specific survival for this cohort. This is the case when events are censored at five and ten years, and with full follow-up [43]. This finding prompts the question of whether these sets remain significant in combination and in the presence of clinicopathological parameters, such as estrogen-status, lymph node involvement, and tumor size and grade. Of particular interest is that the univariate results suggest that at least one of the stromal sets, that containing *LAMA2*, may be prognostic for estrogen-negative breast cancer. If this proves to be truly the case, then stromal factors might be usefully incorporated into the next generation of breast cancer classifiers/disagnostic functions since first-generation signatures fail to discriminate good from poor prognosis for women with estrogen receptor-negative disease.

Table 2 records the results from a series of univariate and multivariate Cox models for this dataset. The object is to discern whether the predictors in the models (rows) add independent prognostic value for the population of samples indicated by the columns. Several of the multivariate models appear twice, once with one predictor in bold font and a second time with a second predictor in bold, e.g., “**proliferation** + decorin” and “**decorin** + proliferation”. The entries in the table are the z values of the corresponding predictor. Z values with a *p* value less than 0.05 appear in bold. The purpose of the table is to provide a summary view of the sets or clinical measures that add prognostic information, and in which combinations, for the cohort restricted to estrogen receptor-positive samples, and for the cohort restricted to estrogen receptor-negative samples. Each entry in the table is taken from a Cox proportional hazards model which is presented in full as a table in File S1.

**Table 2:** Cox proportional hazards models for the UPPSALA cohort.

Cox proportional hazards models	Uppsala 202 ER+full follow-up	Uppsala 202 ER+censored@5 years	Uppsala 34 ER-full follow-up	Uppsala 34 ER-censored@5 years
**Proliferation**	**3.71**	**3.36**	1.31	0.24
**proliferation** + clinical	**2.67**	**2.36**		1.12
**proliferation** +decorin	**2.17**	**2.15**	0.43	0.69
**proliferation** + decorin + clinical	1.37	1.44	0.03	0.57
**proliferation** + laminin	**2.06**	**2.38**	−0.55	−0.52
**proliferation** + laminin + clinical	1.72	**2.11**	−0.79	−0.02
**Decorin**	**−4.36**	**−3.75**	−1.73	−1.19
**decorin** + clinical	**−3.65**	**−2.89**		−1.56
**decorin** + proliferation	**−2.97**	**−2.44**	−0.99	−0.44
**decorin** + proliferation + clinical	**−2.74**	**−2.09**	−1.44	−1.1
**laminin**	**−3.43**	**−2.5**	−1.92	−1.74
**laminin** + clinical	**−2.97**	−1.16		**−1.97**
**laminin** + proliferation	−1.35	−0.38	−1.56	−1.47
**laminin** + proliferation + clinical	−0.53	0.47	**−2.09**	−1.77

Column labels indicate subsets of samples and follow-up period, e.g., “ER+@5” stands for estrogen-receptor positive samples with events censored at five years. Rows specify the predictors in a Cox proportional hazards model. Table entries report the z value of the first predictor of the model in the corresponding row for the samples in the corresponding column. Entries with *p-*values less than 0.05 appear in bold.

Focusing on the models for the estrogen-positive and estrogen-negative subsamples the essential finding for the Uppsala cohort is that proliferation and stromal-decorin are independently prognostic for estrogen-positive tumors, but not for estrogen-negative ones, whereas stromal-laminin is either significant or marginally significant for the estrogen-negative subpopulation. In more detail, for the 202 estrogen-positive samples in the Uppsala cohort, of the four clinical measures (age, grade, tumor size and nodal status), only size and nodal status remain significant in multivariate models selected by backwards elimination. For these estrogen receptor-positive samples, proliferation is prognostic at full follow-up and with events censored at five years, as a single predictor and in combination with stromal-decorin and with clinical variables, but not with both. In contrast, for these estrogen receptor-positive samples stromal-decorin is prognostic at full follow-up and with events censored at five years, as a single predictor and in combination with proliferation and clinical descriptors. For the 34 estrogen-receptor negative samples, only stromal-laminin is significant, and only with censoring at five years and in combination with clinical variables, though stromal-laminin is marginally significant as a single predicator for full follow-up and with events censored at five years. Among the Cox models summarized in Table 2, the two models extracted to Tables 3 and 4 are of particular interest. The domain for the first is estrogen receptor-positive samples, using as predictors proliferation, stromal-decorin, and clinical descriptors (nodal status and tumor size). The domain for the second is estrogen receptor-negative samples, using as predictors proliferation, stromal-laminin and nodal status and tumor size. The stromal sets in these two models are significant while the proliferation set is not, as reflected in the hazard ratios and *p*-values.

**Table 3:** Multivariate Cox proportional hazards model with stromal-decorin for UPPSALA estrogen receptor-positive samples.

UPPSALA estrogen receptor-positive samples (n = 202) with full follow-up						
	log(HR)	HR	Z	p	95CI-lower	95CI-upper
nodal status	1.15	3.17	3.755	0.0001	1.73	5.8
tumor size	0.02	1.02	2.324	0.02	1.004	1.05
proliferation	0.38	1.46	1.37	0.17	0.84	2.53
stromal-decorin	−0.67	0.5	−2.748	0.005	0.31	0.82
	likelihood ratio test = 47, 4 df, p = 1.41E-09					

**Table 4:** Multivariate Cox proportional hazards model with stromal-laminin for UPPSALA estrogen receptor-negative samples.

UPPSALA Estrogen receptor-negative samples (n = 34) with full follow-up						
	log(HR)	HR	Z	p	95CI-lower	95CI-upper
nodal status	1.2	3.33	1.23	0.21	0.48	22.8
tumor size	0.05	1.06	0.915	0.36	0.93	1.2
proliferation	−1.71	0.17	−0.797	0.42	0.008	12.26
stromal-laminin	−2.6	0.07	−2.099	0.03	0.006	0.84
	likelihood ratio test = 8.58, 4 df, p = 0.07					

In summary, for the Uppsala cohort, the regressions, supplemented by the visual check of the partitions, provide evidence that

- In multivariate models with proliferation, stromal-decorin is an independent predictor of disease specific survival.
- While there are too few events in the Uppsala cohort to be confident of the pattern, visual inspection of the data suggest that the stromal-laminin gene set may be prognostic of disease specific survival of estrogen-negative patients, which is not the case for the proliferation set.

### Stockholm

Our first attempt to confirm the UPPSALA results on a second dataset was somewhat disappointing. The STOCKHOLM dataset provides evidence that stromal-decorin is prognostic for estrogen receptor-positive samples and stromal-laminin for the estrogen receptor-negative samples, but in both cases the association does not reach statistical significance. The STOCKHOLM dataset consists of 159 breast cancer patients treated at the Karolinska Hospital between January 1994 and December 1996 [49]. As an unselected, population-based study of Swedish women it resembles the UPPSALA cohort. The logged and normalized Affymetrix HG-U133A expression data is available at GEO as series GSE1456, along with clinical descriptions (version 2, revised June 12, 2009). The GEO expression data does not include estrogen-status, though the overall proportion of estrogen receptor-negative samples to estrogen receptor-positive samples (29/130) can be inferred from a summary table in the published article [49]. To assemble ER+ and ER- groups we split the 159 samples at the 18.2 quantile using the estrogen-related gene set described earlier. As summarized in Table 5, the same series of Cox proportional hazards models as in the UPPSALA analysis reveals that:

**Table 5:** Cox proportional hazards models for the STOCKHOLM cohort.

	Stockholm130ER+ full	Stockholm130ER+ @5	Stockholm29ER- full	Stockholm 29ER- @5
**proliferation**	**3.46**	**2.79**	0.83	0.75
**proliferation** + clinical	**2.29**	1.81	0.61	0.104
**proliferation** + decorin	**3.14**	**2.78**	−0.76	0.49
**proliferation** + decorin + clinical	**2.01**	1.87	−0.31	0.13
**proliferation** + laminin	**2.6**	**2.1**	−0.64	−0.34
**proliferation** + laminin + clinical	1.73	1.35	−0.76	−0.54
**decorin**	−1.55	−0.88	−0.85	−0.608
**decorin** + clinical	−1.11	−0.44	−0.71	−0.54
**decorin** + proliferation	0.83	1.08	−1.26	−0.02
**decorin** + proliferation + clinical	0.38	0.77	−0.9	−0.24
**laminin**	**−2.39**	−1.91	−1.68	−1.32
**laminin** + clinical	−1.5	−1.21	−1.26	−1.05
**laminin** + proliferation	0.23	0.15	−1.73	−1.22
**laminin** + proliferation + clinical	0.004	−0.03	−1.43	−1.14

Column labels indicate subsets of samples and follow-up period, e.g., “Stockholm130ER+@5” stands for estrogen-receptor positive samples with events censored at five years. Rows specify the predictors in a Cox proportional hazards model. Table entries report the z value of the first predictor of the model in the corresponding row for the samples in the corresponding column. Entries with *p-*values less than 0.05 appear in bold.

- Stromal-decorin is prognostic as a univariate predictor of disease specific survival for the dataset as a whole with full follow-up, but only marginally so for the estrogen-receptor positive samples, (z = −1.55, 95CI 0.38–1.11, *p* = 0.11).
- For the estrogen-negative subset only stromal-laminin is prognostic, and then only marginally so (z = −1.68, 95CI 0.1–1.18, p = 0.09).

Inspecting the multivariate models for the STOCKHOLM data there is not strong evidence that the stromal-decorin gene set adds prognostic information to proliferation. The stromal-laminin set may add prognostic value for estrogen-receptor negative samples, but at a level that falls below statistical significance.

The difference in the findings for the two datasets, UPPSALA and STOCKHOLM, might be ascribed to differences in the number of samples or to the composition of the samples in terms of clinical descriptors. With only 130 and 29 samples in the ER+ and ER− groups, it could well be the case that the multivariate Cox proportional hazards models built for the STOCKHOLM cohort lack the statistical power to discern the relationship between the stromal sets and proliferation observed in the UPPSALA data. Alternatively, or in addition, though in both UPPSALA and STOCKHOLM studies patients were accrued consecutively and in an unselected manner from the population, a comparison of the clinical descriptions of the women may show a bias, e.g., a disproportionate number of older or more progressed cases. With this possibility in mind, we investigated the association between the stromal gene sets and clinical descriptors in the UPPSALA data. Of the five clinical variables available (histologic grade, ER-status, PgR-status, age, lymph node-status and tumor size) after backward elimination only lymph node-status and tumor size remained significant in the multivariate Cox models with proliferation and stromal-decorin or stromal-laminin (File S1).

### Lymph Node-status

**Figure 1:**
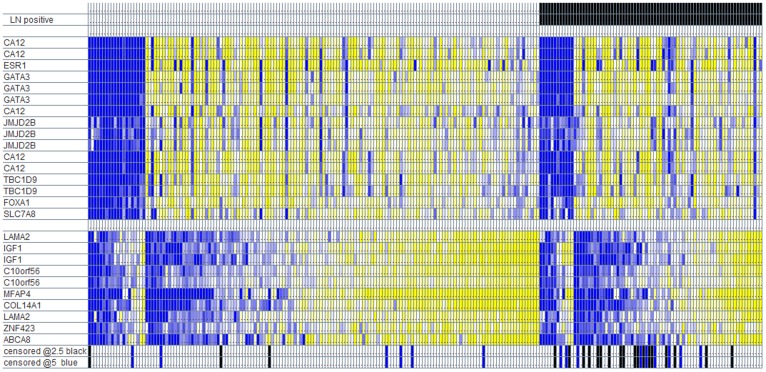
Uppsala samples partitioned by lymph node status and estrogen-status, ordered on stromal-laminin gene expression. Yellow signifies up-regulation; blue signifies down-regulation. Rows represent probe sets on the Affymetrix HG U133A platform. Black bars record Breast Cancer Specific Survival events censored at 2.5 years. Blue bars record BCSS events that occur between 2.5 and 5 years.

Investigating the association between stromal set expression and lymph node status in the estrogen receptor-positive and estrogen receptor-negative subcohorts in the UPPSALA data, it is amply clear that the stromal sets add prognostic value to lymph node-status. This is visibly apparent in Figure 1 which arranges the UPPSALA patients by lymph node- and estrogen-status, then orders the samples by stromal-laminin expression. As evidenced by how the attached Disease Specific Survival events (censored at 2.5 and 5 years) cluster at the low end of stromal-laminin expression, it would appear that stromal-laminin adds considerable prognostic information to lymph node-status, in particular to the subset of samples that are lymph node-positive. The same is the case for stromal-decorin. If the prognostic value of the stromal sets depend on the presence (and relative proportion) of lymph node-positive samples in a dataset, then the discrepancy in the results between the UPPSALA and STOCKHOLM cohorts might be explained by a difference in the proportion of lymph node-positive patients. Unfortunately, though the overall number of lymph node-positive samples in the UPPSALA dataset can be inferred from a summary table in [49], the lymph node status of the individual samples is not supplied in the public version of the dataset.

Pursuing the notion that the prognostic value of the stromal sets might be confined to lymph node-positive samples, we examined two additional datasets, SAN FRANCISCO [50] and MAINZ [32], selected because they differ substantially in the proportion of lymph node positive tumors, one with an abundance of lymph node positive samples, and a second with none at all. For both of these cohorts we built the same series of Cox proportional hazards models as for the UPPSALA and STOCKHOLM data.

### San Francisco

The third dataset consists of 118 tumor samples collected at the University of California San Francisco and Pacific Medical Center between 1989 and 1997 [50]. It is enriched in lymph node-positive samples (66 of 118), and in comparison to the UPPSALA cohort contains a greater proportion of samples with larger tumor size and higher histologic grade. The Affymetrix HG-U133A expression data is downloadable from ArrayExpress [51], accession number E-TABM-158. One sample lacks Distant Metastasis Free Survival (DMFS) event and time, leaving 117 samples for survival analysis. Under backwards elimination with a cutoff *p*-value of 0.1, none of the clinical predictors (age, nodal-status, histologic grade, tumor size or sizing) proved significant in multivariate models. The Cox models summarized in Table 6 show that:

**Table 6:** Cox proportional hazards models for the SAN FRANCISCO cohort.

	SanFrancisco74ER+full	SanFrancisco74ER+@5	SanFrancisco43ER- full	SanFrancsico43 ER-@5
**proliferation**	**2.29**	1.19	0.24	0.76
**proliferation** + clinical				
**proliferation** + decorin	1.77	0.82	−0.09	0.86
**proliferation** + decorin + clinical				
**proliferation** + laminin	0.89	0.29	0.608	0.51
**proliferation** + laminin + clinical				
**decorin**	**−2.92**	**−2.23**	−0.88	0.37
**decorin** + clinical				
**decorin** + proliferation	**−2.54**	**−2.08**	−0.86	0.54
**decorin** + proliferation + clinical				
**laminin**	**−2.7**	−1.65	0.62	−0.87
**laminin** + clinical				
**laminin** + proliferation	−1.93	−1.28	0.84	−0.69

Column labels indicate subsets of samples and follow-up period, e.g., “SanFrancisco74ER+@5” stands for estrogen-receptor positive samples with events censored at five years. Rows specify the predictors in a Cox proportional hazards model. Table entries report the z value of the first predictor of the model in the corresponding row for the samples in the corresponding column. Entries with *p-*values less than 0.05 appear in bold.

- Stromal-decorin is a significant predictor in multivariate models with proliferation for the the estrogen receptor-positive subset with full follow-up and with dmfs events censored at five years.
- The stromal-laminin gene set is prognostic as a single predictor for the estrogen-positive subset with full follow-up.
- None of the three gene sets, proliferation, stromal-decorin, or stromal-laminin, is prognostic for the estrogen receptor-negative cohort, in either univariate or multivariate models.

Essentially the SAN FRANCISCO data confirms the finding in UPPSALA regarding the prognostic value of stromal-decorin for estrogen receptor-positive subcohort, but it does not confirm the finding regarding the value of stromal-laminin for the estrogen receptor-negative sub-cohort.

### Mainz

Further pursuing the possible association between the prognostic value of the stromal sets and lymph node-status, to set up a contrast with the SAN FRANCISCO data, we built our series of Cox models for a dataset in which the samples are exclusively lymph node-negative. Like the UPPSALA cohort, the 200 samples in the MAINZ dataset represent a population-based consecutive series, in this case accrued at Mainz between 1988 and 1998 [32]. The normalized Affymetrix HG-U133A expression data is available at GEO as series GSE11121, which includes clinical variables for histological grade and tumor size, but not for estrogen-status, though the overall proportion of estrogen-negative to estrogen-positive patients is reported in the original article: (44 (22%) estrogen-negative and 156 (78%) estrogen-negative. To form the estrogen receptor-positive and estrogen receptor-negative subpopulations we ordered the samples on the estrogen-related gene set, dividing at the 22^nd^ quantile. The endpoint for this dataset was Distant Metastasis Free Survival. The results for the MAINZ data, as summarized in Table 7, show that:

**Table 7:** Cox proportional hazards models for the MAINZ cohort.

Cox proportional hazards models	Mainz 156ER+ full	Mainz 156ER+ @5	Mainz 44ER- full	Mainz 44ER- @5
**proliferation**	**4.22**	**5.3**	**2.68**	**2.65**
**proliferation** + clinical	**3.19**	**4.32**		
**proliferation** + decorin	**2.86**	**3.61**	**2.33**	**2.51**
**proliferation** + decorin + clinical	**2.33**	**3.16**		
**proliferation** + laminin	**3.09**	**3.68**	**3.24**	**3.28**
**proliferation** + laminin + clinical	**2.43**	**3.21**		
**decorin**	**−2.87**	**−3.38**	−1.27	−1.04
**decorin** + clinical	**−2.31**	**−2.77**		
**decorin** + proliferation	−1.67	**−2.32**	0.33	0.63
**decorin** + proliferation + clinical	−1.57	**−2.03**		
**laminin**	**−2.23**	**−2.63**	−1.27	−0.01
**laminin** + clinical	−1.77	**−2.09**		
**laminin** + proliferation	−0.56	−1.06	**1.99**	**2.18**
**laminin** + proliferation + clinical	−0.63	−0.79		

Column labels indicate subsets of samples and follow-up period, e.g., “Mainz156ER+@5” stands for estrogen-receptor positive samples with events censored at five years. Rows specify the predictors in a Cox proportional hazards model. Table entries report the z value of the first predictor of the model in the corresponding row for the samples in the corresponding column. Entries with *p-*values less than 0.05 appear in bold.

- For the estrogen receptor-positive subset, stromal-decorin adds independent prognostic information to proliferation with dmfs events censored at five years, and marginally with full follow-up.
- For the estrogen receptor-negative subset, stromal-laminin in combination with proliferation is prognostic of dmfs with events censored at five years and for full follow-up.

The results for the MAINZ cohort, in which all samples are lymph node-negative, suggests strongly that the prognostic value of the stromal sets is not confined to lymph node-positive tumors. Therefore, the contrast in the results for the UPPSALA and STOCKHOLM cohorts is likely due to some other factor. Comparing the clinical descriptions of the samples in the four datasets, the most striking difference is the distribution of patients by age, as tabulated in Table 8. The principal contrast is between the UPPSALA and MAINZ cohorts, with median age of 64 and mean age of 60, respectively, and the STOCKHOLM and SAN FRANCISCO datasets, with median ages of 56 and 51. Also telling is the fact that 46% of the SAN FRANCISCO patients are 50 years of age or younger and perhaps premenopausal. The proportion of younger and premenopausal women in the STOCKHOLM dataset cannot be determined, but, given the median age of those patients, is perhaps significantly larger than in the UPPSALA cohort where only 22% are less than fifty years of age. Hence, the difference in results for UPPSALA compared to STOCKHOLM may be attributed to the larger proportion of older women in the UPPSALA cohort. If the stromal sets are prognostic for older women but not for younger, this would account for a separate finding reported in [43], namely that the stromal-decorin set is not prognostic for the TRANSBIG cohort [52], which has a median age of 49, with 76% younger than 55, and only 1% older than 70, as reported in [32].

**Table 8:** Age distribution for five datasets.

dataset	accession	samples	median age*	< = 50 years
UPPSALA	GSE3494	251	64+−14	22%
MAINZ	GSE11121	200	60+/−12	35%
STOCKHOLM	GSE1456	159	56+/−14	NA
SAN FRANCISCO	E-TABM-158	118	51+/−15	46%
TRANSBIG	GSE7390	198	46+/−7	69%

Median age for UPPSALA, STOCKHOLM, SAN FRANCISCO, and TRANSBIG cohorts. Mean age for MAINZ. Percentage of samples 50 years of age or younger. Source for median ages = [57]. Source for percentage samples less than 51 = [58].

To test whether the prognostic value of the stromal sets is dependent on age, we split the UPPSALA cohort at age sixty and built Cox proportional hazards models to test the two propositions:

1. That the stromal-decorin gene set adds prognostic information to proliferation for estrogen receptor-positive women with invasive breast cancer.
2. That the stromal-laminin gene set is prognostic for estrogen receptor-negative women with invasive breast cancer.

For the estrogen receptor-positive women the models recorded in Table 9 show that stromal-decorin is significantly prognostic independently of proliferation for the older women (>60 years) in the UPPSALA dataset, but not for the younger women (<60 years). Testing the second proposition regarding the prognostic value of stromal-laminin for estrogen receptor-negative women in the UPPSALA cohort is a challenge given the small number of samples and events. Nevertheless, there is slight evidence that stromal-laminin is prognostic for the older women, but not for the younger (Table 10).

**Table 9:** Cox proportional hazards models for UPPSALA estrogen receptor-positive older (>60) and younger (<60) women.

	HR	95CI(lower-upper)	z score	*p* value
proliferation	2.27	0.91–5.65	1.76	0.07
stromal-decorin	0.48	0.25–0.91	−2.22	0.02
	Likelihood Ratio Test = 9.51, 2 df, *p* = 0.008			
	**HR**	**95CI(lower-upper)**	**z score**	***p*** ** value**
proliferation	2.19	0.63–7.61	1.24	0.35
stromal-decorin	0.57	0.17–1.86	−0.92	0.35
	Likelihood Ratio Test = 5.94, 2 df, *p* = 0.05			

Upper model: UPPSALA estrogen receptor-positive, older women (>60 years of age) n = 117, 19 Breast Cancer Specific Survival events censored @ 5 years.

Lower model: UPPSALA estrogen receptor-positive, younger women (<60 years of age) n = 83, 13 Breast Cancer Specific Survival events censored @ 5 years.

Summarizing results across the four datasets:

- In three of the four cohorts (UPPSULA, SAN FRANCISCO, and MAINZ), for estrogen-positive patients the stromal-decorin gene set adds independent prognostic information in multivariate models that include proliferation expression and clinicopathological variables.
- In two datasets (UPPSALA and MAINZ) and marginally in a third (STOCKHOLM), for estrogen-negative patients the stromal-laminin gene set adds prognostic value.

These results may be conditioned on age, holding for older, but not younger women.

**Table 10:** Cox proportional hazards models for UPPSALA estrogen receptor-negative older (>60) and younger (<60) women.

	HR	95CI(lower-upper)	z score	p value
stromal-laminin	0.084	0.008–0.88	−2.06	0.03
	Likelihood Ratio Test = 6.37, 1 df, p = 0.01			
	**HR**	**95CI(lower-upper)**	**z score**	**p value**
stromal-laminin	2.13	0.13–34.4	0.53	0.59
	Likelihood Ratio Test = 0.28, 1 df, p = 0.59			

Upper model: UPPSALA estrogen receptor-negative, older women (>60 years of age) n = 20, 3 Breast Cancer Specific Survival events censored @ 5 years.

Lower model: UPPSALA estrogen receptor-negative, younger women (<60 years of age) n = 10, 1 Breast Cancer Specific Survival event censored @ 5 years.

## Discussion

The expression of the genes in the stromal-decorin and stromal-laminin sets appear to be switch-like. For the women most at risk of metastasis and death the expression of these genes is essentially absent. Why decorin expression is lost is unknown, but like the loss of laminin and caveolin expression, it may stem from changes in, and ultimately from the extinction of, the myoepithelial cell layer. As SAGE studies of cell type and cell state document, laminin and caveolin are lost first. Whereas laminin and caveolin are expressed by normal myoepithelial cells, and subsequently lost by DCIS-involved myoepithelial cells, the reverse is the case for decorin. That is, *DCN* is expressed by the cancer-transformed myoepithelial cells, and not by the normal [53], [54]. The up-regulation of decorin might be part of a stromal host response similar to foreign body response which deposits a wall of ECM around an offending object. If that defensive response succeeds in encapsulating the tumor while still small, the tumor may never attain detectable size [55]. In that case the myoepithelial layer remains intact, serving not only as a physical barrier with the BM (the first line of defense), but continuing to produce, in sync with fibroblasts, the right mixture of matricellular proteins, fibrillar collagens, etc. needed for the dynamic maintenance of the second line of defense, the interstitial ECM reinforced by the host stromal response. The loss of the myoepithelial layer might sufficiently alter the mix of gene expression and products such that, the continuing desmoplastic reaction assembles an inferior decorin-deficient matrix. The consequence is that what had functioned as a barrier and container is progressively transformed into a gateway and facilitating substrate for the advancing vanguard of infiltrating tumor cells. In short, loss of decorin expression is perhaps both indicator and causal factor of this change.

As we have been most recently reminded by A. Bergamaschi (personal communication), physiological differences in the structure and stiffness of breast tissue is associated with aging. Here at the intersection of the biology of tumor progression and the biology of ageing, an emerging message from the data analysis is that the protective value of the expression of the stromal sets holds for older women, but not for younger. That is, older women with breast cancer who are capable of mounting a robust stromal response are those who are most likely to survive their disease. Monitoring that response as mirrored in the expression of the stromal-decorin and stromal-laminin sets may be strategic for patient management in this cohort.

After a decade of development, there has been an increasingly urgent call for gene signatures that do not rely exclusively on proliferation to discriminate good from poor prognosis [22]. Incorporating the stromal sets takes a step in a direction which may lead to a signature that is both grounded in the biology of the disease and more accurate in identifying patients at greatest risk.

## Materials and Methods

The stromal and proliferation gene sets used as predictors in the multivariate Cox proportional hazards models were induced by the partition-based algorithm described in [43] as applied to the Uppsala dataset [44]. For the derivation of the gene sets with a worked example, see Supplemental Methods in File S2, which describes instances of the stromal-decorin gene set (Table S1) and of all gene sets detected in the UPPSALA data (Table S2).

Univariate and multivariate Cox proportional hazards models with combinations of gene sets and clinicopathological measures were built using the coxph function in the r survival package [56] using backward elimination with a *p*-value cutoff of 0.1 applied to the Affymetrix HG-U133A expression data for four breast cancer datasets designated as: UPPSALA [44], MAINZ [32], SAN FRANCISCO [50], and STOCKHOLM [49]. The full description and result for each model is tabulated in File S1.

To visually inspect the prognostic value of the stromal genes in combination with proliferation genes and lymph node status we partitioned and stacked heatmaps to provide the equivalent of binary decision trees. These were programmed in Java by the author.

## Supporting Information

Table S1 An Excel workbook that records the gene sets detected by the partition-based algorithm applied to the UPPSALA data with partition size = 129, and tolerance for mismatch = 17.
(XLS)

Table S2 An Excel workbook that records all of the gene sets detected in the UPPSALA data.
(XLS)

File S1 An Excel workbook with twenty-four spread sheets which record Cox proportional hazards models (six worksheets for each of four datasets). Each sheet contains six to twelve or more models grouped by domain (all samples, estrogen-positive samples, estrogen-negative samples) and by follow-up (censored at five years and full follow-up).
(XLS)

File S2 Supplemental Methods. A Word document that describes the derivation of the gene sets.
(DOC)
