# Supplementary material for: Stromal Genes Add Prognostic Information to Proliferation and Histoclinical Markers: A Basis for the Next Generation of Breast Cancer Gene Signatures
Source: PLoS One. 2012 Jun 18;7(6):e37646. doi: 10.1371/journal.pone.0037646 (PMC3377707; doi:10.1371/journal.pone.0037646)
Supplement: File S2 — Supplemental Methods. A Word document that describes the derivation of the gene sets. (DOC) [file pone.0037646.s004.doc]

Supplemental Methods

To test whether the stromal gene sets add prognostic information independent of proliferation and clinical variables, we built Cox proportional hazards models using the r survival package [1]. Essentially, proliferation and the stromal gene sets, along with selected clinical measures such as lymph node-status are regressed, in different combinations, on survival data for each of four Breast Cancer datasets (GSE3494[2], GSE11121[3], E-TABM-158[4], GSE1456[5]). Models were built for each of the datasets as a whole with survival events censored at five years, and with full follow-up. Additional models were built for the estrogen receptor-positive and estrogen receptor-negative samples, again with events censored at five years and with full follow-up. The results are collected as spread sheets in the excel workbook in File S1.

### Derivation of the gene sets

What may be of principal interest is the derivation of the gene sets which we have labeled: “stromal-decorin” , “ stromal-laminin”, “proliferation” , and “estrogen”. These are sets of genes (probesets) which have been found to partition the bulk Breast Cancer tumor samples collected in a number of gene expression based studies, including the four used in this article. To describe the algorithm and what it detects it may be useful to provide the worked example of how the stromal-decorin set was induced from the UPPSALA data.

### Key intuition

We start from the observation that a number of genes partition a collection of tumor samples in the same or nearly the same way. A pattern-matching algorithm finds these redundant patterns in a matrix of expression values and outputs lists of genes that support these partitions. One of the largest of these lists, or sets, contains *ESR1* as described in the article. Others are composed of genes that can be readily associated with cell types or biological mechanisms, including T-lymphocytes, B-cells, mast cells, hemoglobin, adipocytes … For example, In the UPPSALA data the algorithm finds:

CD48, CD37, LCK, CD2, TRAC, TRA@ …

IGLJ3, IGHM, IGHG3, IGL, IGL@, TNFRSF17 …

TPSAB1, TPSB2, TPSAB1, TPSAB1, TPSAB …

HBA1, HBA1, HBB, HBA2, HBA2, HBG1 …

LPL, LPL, FABP4, PLIN, ADIPOQ, ADHIB, GOS2, CIDEC …

For this dataset, the algorithm returns about thirty-five such sets. As enumerated in the 2010 BMC Genomics article [6], the algorithm finds essentially these same sets in the data collected by each of several breast cancer studies, which suggests that the partitioning patterns constitute a conserved feature of the domain from which this data has been sampled.

### Essential steps

The algorithm repeatedly calls a matching function that treats each gene as a pattern to be compared to that of each of the other genes. The essential steps include:

1. Set up a schedule for the two parameters that control the matching function, namely the size of the partition (number of samples), and the number of mismatches to be tolerated when determining whether partitions of this size are the “same”.

2. For each combination of these parameters (size s, and tolerance t), call the matching function which finds rows (genes) that induce the “same” partition. A partition is defined by the subset of samples (columns) indexed by the s largest expression values for each gene, allowing for up to t mismatches.

3. Remove redundancies in the lists of sets returned by the calls to the matching function. Return the reduced list of distinct gene sets.

### A worked example

A single call to the matching function can serve as an example of the essential step in the algorithm. In the UPPSALA data the stromal-decorin set is first detected once the size parameter has been stepped to 135 and tolerance incremented to 19. At this combination of size and tolerance 14 gene sets are detected as listed in Table 1, which records just the first several genes for each set. (The full table appears as Table S1). The gene sets found at this call to the matching function range in size from the six in the stromal-decorin set in row 13 to the 53-gene set in the first row, which is an instance of the “proliferation” set. Other sets include a stromal set in row 4, which closely resembles West et al.’s Desmoid-Type Fibromatosis signature [7], and a set in row 6 composed of Affymetrix endogenous controls.

| H2AFZ | TOP2A | TOP2A | RRM2 | BIRC5 | KIAA0101 | FOXM1 | CCNB2 | OAS1 | UBE2C |
| --- | --- | --- | --- | --- | --- | --- | --- | --- | --- |
| CCL5 | HLA-DPB1 | LAPTM5 | LAPTM5 | DHPS | CSF1R | CD53 | SLCO2B1 | CXCL9 | HLA-DMB |
| POU2AF1 | TNFRSF17 | 209138 | 211633 | 211634 | 211635 | 211637 | 211640 | 211641 | 211643 |
| SPARC | LUM | COL3A1 | COL1A2 | FBN1 | ADAM12 | COL5A1 | CSPG2 | OMD | OMD |
| GPX3 | LPL | LPL | FABP4 | AKR1C1 | PLIN | CD36 | ADIPOQ | ADH1B | ADH1B |
| AFFX-BioB- | AFFX-BioC | AFFX-BioC | AFFX-Bio | AFFX-BioDn- | AFFX-CreX | AFFX-CreX | AFFX-r2-Ec | AFFX-r2- | AFFX-r2 |
| KLHL24 | PDE4C | 207730 | 208246 | ZNF160 | RIOK3 | 215600 | LOC152719 | KNS2 | 216524 |
| PSMB9 | HLA-F | HCP5 | HLA-B | PSMB8 | HLA-G | HLA-G | HLA-C | HLA-B | HLA-C |
| HBA1 | HBB | HBA1 | HBB | HBA1 | HBA2 | HBA2 | HBB | HBA2 |  |
| ACTG1 | ACTG1 | ACTG1 | ACTG1 | ACTG1 | ACTG1 | ACTG1 | ACTG1 |  |  |
| STAT1 | UBE2L6 | CXCL10 | STAT1 | CXCL11 | CXCL11 | AFFXHUM | AFFX-HUM |  |  |
| TPSAB1 | TPSB2 | TPSAB1 | TPSAB1 | TPSAB1 | TPSAB1 | TPSAB1 |  |  |  |
| DCN | FBLN1 | FBLN1 | DCN | DCN | DCN | GLT8D2 |  |  |  |
| GGT1 | 208284 | GGT1 | GGTLA4 | GGT1 | GGT2 |  |  |  |  |

Table 1. A portion of the list of gene sets returned for the UPPSALA data at size=129, and tolerance=17. For the full table see Table S1.

Altogether for the UPPSALA data the algorithm makes 220 calls to the matching function, each of which produces a list of sets like that in Table 1. Collecting and reducing this output yields 35 distinct gene sets as enumerated in the 2010 BMC Genomics article. For the purpose of viewing the full output of the algorithm as applied to the UPPSALA data, labels are assigned to the gene sets, generally a prominent gene within the set, e.g., “HBA1” for hemoglobin and TPSAB1 for a set of genes associated with mast cells. We also use short labels such as “immune(0)” and “immune(1)”, which stand for T-lymphocyte and immune globulin or B-cell. Applied to the UPPSALA dataset, the first ten calls to the matching function return the sets listed in Table 2, where the first two columns record size and tolerance, and the gene sets are arranged by size. (The full output for this dataset is available as Supplemental Table S2).

| 20 | 3 | immune(0) | 26 | HBA1 | 9 | immune(1) | 8 | immune(1) | 8 | TPSAB1 | 7 |  |  |  |  |
| --- | --- | --- | --- | --- | --- | --- | --- | --- | --- | --- | --- | --- | --- | --- | --- |
| 21 | 4 | immune(0) | 78 | immune(1) | 58 | HBA1 | 10 | AFFX-BioC | 8 | TPSAB1 | 7 | stromal(0) | 7 | erbb2 | 6 |
| 22 | 4 | immune(0) | 60 | immune(1) | 42 | HBA1 | 9 | mmune(5) | 8 | TPSAB1 | 7 | AFFX-BioC | 6 |  |  |
| 23 | 4 | immune(0) | 61 | immune(1) | 40 | HBA1 | 9 | immune(5) | 8 | AFFXBioC | 8 | TPSAB1 | 7 | erbb2 | 6 |
| 24 | 4 | immune(0) | 56 | immune(1) | 20 | AFFX-BioC | 12 | immune(1) | 9 | TPSAB1 | 7 | HBA1 | 6 |  |  |
| 25 | 5 | immune(0) | 105 | immune(1) | 55 | AFFX-BioC | 14 | erbb2 | 10 | TPSAB1 | 7 | adipose | 7 | HBA1 | 6 |
| 26 | 5 | immune(0) | 92 | immune(1) | 58 | AFFX-BioC | 14 | HBA1 | 10 | erbb2 | 9 | TPSAB1 | 7 |  |  |
| 27 | 5 | immune(0) | 74 | immune(1) | 53 | AFFX-BioC | 13 | HBA1 | 9 | erbb2 | 9 | TPSAB1 | 7 |  |  |
| 28 | 5 | immune(0) | 65 | immune(1) | 53 | AFFX-BioC | 13 | HBA1 | 9 | TPSAB1 | 7 |  |  |  |  |
| 29 | 6 | immune(1) | 185 | AFFX-BioC | 16 | erbb2 | 15 | HBA1 | 10 | TPSAB1 | 7 | immune(2) | 7 |  |  |

Table 2. A portion of the full list of gene sets detected in the UPPSALA data. The first two columns record size and tolerance. For the full output see Table S2.

Extracting just the instances of the stromal-decorin set from the full output yields the sets in Table 3, the first two columns of which, again, record the size and tolerance settings at which stromal-decorin is detected.

| 129 | 17 | DCN | FBLN1 | FBLN1 | DCN | DCN | DCN | GLT8D2 | .......... | .......... | .......... | .......... | .......... |  |
| --- | --- | --- | --- | --- | --- | --- | --- | --- | --- | --- | --- | --- | --- | --- |
| 145 | 16 | DCN | FBLN1 | FBLN1 | DCN | DCN | DCN | GLT8D2 | CTSK | .......... | .......... | .......... | .......... |  |
| 150 | 16 | DCN | FBLN1 | FBLN1 | DCN | DCN | DCN | .......... | .......... | .......... | .......... | .......... | .......... |  |
| 203 | 11 | DCN | FBLN1 | FBLN1 | DCN | DCN | DCN | .......... | .......... | PRRX1 | SPON1 | .......... | .......... |  |
| 205 | 11 | DCN | FBLN1 | FBLN1 | DCN | DCN | DCN | .......... | .......... | .......... | SPON1 | LUM | SPON1 |  |

Table 3. The five instances of the stromal-decorin gene set detected in the UPPSALA data.

To construct the continuous predictor of stromal-decorin used in the Cox proportional hazards models, we arbitrarily selected from the stromal-decorin probe sets those that appear in at least half of the instances, which in this case yields:

DCN, FBLN1, FBLN1, DCN, DCN, DCN

The stromal-laminin, “proliferation”, and “estrogen” gene sets were derived in the same way.

### Stability of the gene sets

A question arises regarding variability/stability of the gene sets across cohorts or experiments. The variability in the composition of the individual “versions” of a gene set can be illustrated with the case of the ERBB2-containing gene set. Applied to four Affymetrix HU133A and U133Aplus2.0 Breast Cancer datasets, the pattern-matching algorithm detects four specific instances of this gene set. Extracting and reducing these sets as in the stromal-decorin example yields four “ERBB2” gene sets:

STARD3 GRB7 ERBB2 GSDML PERLD1 PERLD1 (EMC 286 GSE2034[8])

STARD3 GRB7 ERBB2 ERBB2 PERLD1 PERLD1 (UPPSALA GSE3494[2])

STARD3 GRB7 ERBB2 PERLD1 c17orf37 (Sabatier GSE21653[9])

STARD3 GBR7 ERBB2 PERLD1 PERLD1 PPARBP CRKRS (TRANSBIG GSE7390[10])

While these lists differ, in terms of the orderings they induce on the samples in the respective datasets, they are effectively interchangeable.

### Motivation

The motivation behind the partition-based approach stems from frustration with Hierarchical Clustering (HCL), which is currently the default method for discovering structure in microarray expression data. Some of the limitations of Hierarchical Clustering can be managed by such best practices as that of using resampling methods to assess the stability of the clusters defined at different levels in the binary tree [11,12]. Also, the ordering on the terminal leaves, most often handled heuristically[13], can be optimized[14,15]. But additional problems remain, the most fundamental of which concerns what can be captured and represented in a binary tree *per se*. When applying HCL to tumor samples, large, highly correlated sets of genes will dominate the clustering solution. For Breast Cancer data, this is the case for a set of genes associated with estrogen, as visually documented in a large number of applications of HCL to breast cancer data published over the past decade. The problem concerns the detection and presentation of clustering patterns associated with genes other than those related to estrogen expression. This problem of dominance or occlusion is particularly acute for clusters that may be associated with very small sets of genes. Figure 1 conveys the issue as encountered in the case of the small stromal-decorin set vis-à-vis that of estrogen.


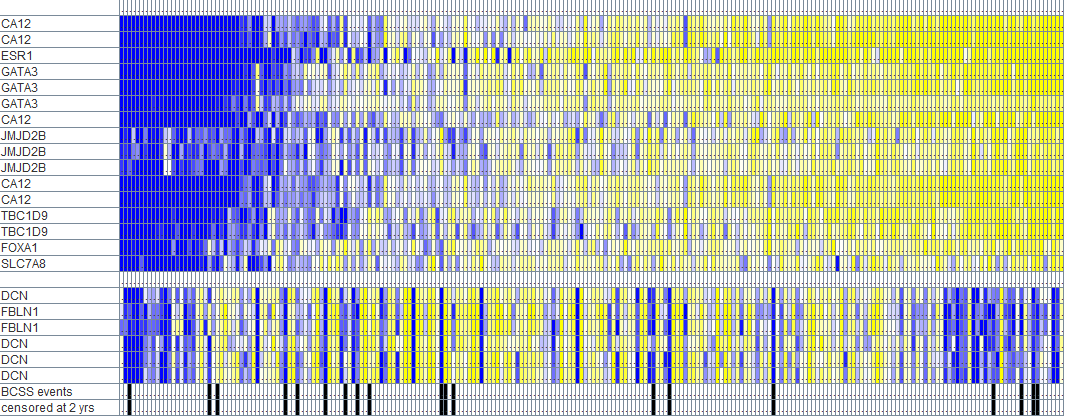


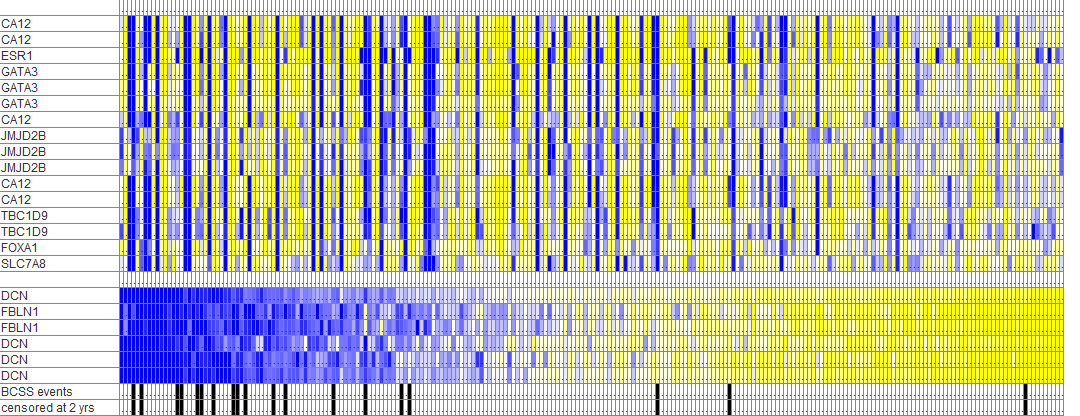


Figure 1. Estrogen and stromal-decorin gene sets as detected in the UPPSALA data. In the upper panel the 251 UPPSALA samples are ordered by estrogen set column sums. In the lower panel, these same samples are ordered by stromal-decorin column sums. Breast Cancer Specific Survival events censored at two years are attached.

If the samples are ordered according to the estrogen signal, the result is as in the upper panel in the figure. If the samples are ordered by the stromal-decorin set, the result is as in the lower panel. It is evident that the two orderings, and the partitions they induce, cannot both be represented under a single ordering of the samples. By virtue of how HCL constructs its hierarchy of trees, it is clear that, for this data, the ordering inherent in the estrogen signal will dominate to the exclusion of that of the stromal signal, and hence the prognostic value of these genes, as displayed in the bottom line in the figure, will be missed.

A formal analysis of this phenomenon is surprisingly difficult, but the sensitivity of HCL to this effect can be studied through simulation by spiking in sub-matrices of varying size and degree of overlap against different levels of background noise. A work-around for this inherent limitation in the standard application of HCL has been proposed which entails removing the signal associated with the dominating cluster and reapplying HCL to the data matrix with that signal removed [16,17]. The partition-based approach represents a more radical solution that does away with tree-building (and its distance matrix) altogether. The clustering or partitioning of the samples, and the sets of genes that induce these partitions, are extracted directly from the matrix of expression values.

As alternative unsupervised methods for finding structure in expression data, Hierarchical Clustering and the partition-based algorithm can be contrasted by how they are used in practice. For HCL the vignette involves applying that algorithm, separately, to the rows and columns of a microarray data matrix, and mapping the rearranged expression values to a red-green color scheme to yield a heatmap [13,18]. The user then searches, informally, for subsets of genes that associate with subsets of samples. Visually these take the form of red patches or rectangles. While the two-way application of HCL to expression data can reveal some of the inherent structure in the data associated with these visual cues, much of the important structure in the data relating subsets of genes to subsets of samples will go undetected. The formal and practical purpose of the partition-based algorithm is to find patterns in the gene expression that distinguish groups of patients. Stated differently, the formal and practical purpose is to find all (and only) the “red rectangles” that a data matrix may contain. An instance of the ERBB2 gene set in the UPPSALA data may serve as an example:


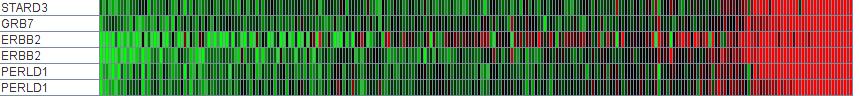


Figure 2. An instance of the ERBB2 gene set as detected in the UPPSALA data at partition size=21 and tolerance for mismatch=4.
